# Health impacts of chemical irritants used for crowd control: a systematic review of the injuries and deaths caused by tear gas and pepper spray

**DOI:** 10.1186/s12889-017-4814-6

**Published:** 2017-10-19

**Authors:** Rohini J. Haar, Vincent Iacopino, Nikhil Ranadive, Sheri D. Weiser, Madhavi Dandu

**Affiliations:** 1 University of California, 3136 College Avenue, Berkeley, CA 94705 USA; 2 Physicians for Human Rights, 256 W 38th Street, 9th Floor, New York, NY 10018 USA; 3 Emory University School of Medicine, 100 Woodruff Circle, Atlanta, GA 30322 USA; 4 Division of HIV, ID and Global Medicine, Department of Medicine, University of California, 533 Parnassus, Box 1031, San Francisco, CA 94143 USA

**Keywords:** Crowd control, Less lethal weapons, Tear gas, Pepper spray, Protests, Demonstrations, 2-chlorobenzalmalonitrile (agent CS), Oleoresin capsicum (agent OC), Pelargonic acid vanillylamide or capsaicin II (PAVA)

## Abstract

**Background:**

Chemical irritants used in crowd control, such as tear gases and pepper sprays, are generally considered to be safe and to cause only transient pain and lacrimation. However, there are numerous reports that use and misuse of these chemicals may cause serious injuries. We aimed to review documented injuries from chemical irritants to better understand the morbidity and mortality associated with these weapons.

**Methods:**

We conducted a systematic review using PRISMA guidelines to identify injuries, permanent disabilities, and deaths from chemical irritants worldwide between January 1, 1990 and March 15, 2015. We reviewed injuries to different body systems, injury severity, and potential risk factors for injury severity. We also assessed region, context and quality of each included article.

**Results:**

We identified 31 studies from 11 countries. These reported on 5131 people who suffered injuries, two of whom died and 58 of whom suffered permanent disabilities. Out of 9261 total injuries, 8.7% were severe and required professional medical management, while 17% were moderate and 74.3% were minor. Severe injuries occurred to all body systems, with the majority of injuries impacting the skin and eyes. Projectile munition trauma caused 231 projectile injuries, with 63 (27%) severe injuries, including major head injury and vision loss. Potentiating factors for more severe injury included environmental conditions, prolonged exposure time, and higher quantities of chemical agent in enclosed spaces.

**Conclusions:**

Although chemical weapons may have a limited role in crowd control, our findings demonstrate that they have significant potential for misuse, leading to unnecessary morbidity and mortality. A nuanced understanding of the health impacts of chemical weapons and mitigating factors is imperative to avoiding indiscriminate use of chemical weapons and associated health consequences.

## Background

The rise in frequency of popular protests in recent years throughout the world is a manifestation of the exercise of the fundamental rights to freedom of expression and peaceful assembly [1]. There are many reports, however, that the frequent use of chemical irritants, commonly referred to as tear gases or pepper sprays, can potentially undermine these freedoms by causing injuries, intimidating communities, and leading to escalations in violence on all sides [2–5].

Chemical irritants are generally expected to cause transient lacrimation, blepharospasm, superficial pain, and disorientation, without permanent injury or death [6, 7]. The first tear gases were developed in the 1920s but, despite the frequency of their use since the 1960s, there has been limited analysis of their mechanisms of injury and potential lethality and longer-term morbidity [8]. Historically, chemical irritants have been considered “nonlethal” or “less lethal” but the intent of temporary irritation may misrepresent the actual health consequences and the impacts of real-world use and misuse of these weapons [8–10].

Chemical irritants are manufactured by many companies around the globe. Historically, most companies were based in the United States, but the past decade has seen the development of manufacturing in Brazil, China, Israel, South Korea, and several other countries [11, 12]. The wide variety of chemical agents, concentrations, unit sizes, and delivery mechanisms used in crowd control complicates full understanding of the effects of these weapons. Research and manufacturer information suggest that chemical irritants can be utilized in a number of ways, but are generally deployed for crowd dispersal or to restrain an individual [13]. Mechanisms of delivery can include sprays or pellets that target specific individuals. Alternatively, canisters, munitions, grenades, and chemical mixtures within water cannons are deployed for crowd dispersal or incapacitation of a large group of people.

Though other chemical agents have been used historically, there are two classes of chemical compounds most commonly used by law enforcement agencies. 2-chlorobenzalmalonitrile (agent CS under military classification) is the most frequently identified active chemical in “tear gas” [14]. Media reports indicate that, in 2013, tear gas was deployed more than 312 times in protests around the world [8]. Though a few countries have significant restrictions on the use of agent CS, many more countries utilize it as their crowd-control weapon of choice [15]. While the effects of CS are considered temporary at low concentrations, higher concentrations have been known to cause permanent injury (primarily to the respiratory system) and death in experimental animal studies as well as anecdotal human exposures [16]. The National Academy of Sciences in the United States does not identify a minimum safe concentration, as even the lowest concentrations can result in “notable discomfort, irritation, or certain asymptomatic, non-sensory but transient effects” [17, 18].

Oleoresin capsicum (agent OC) and its synthetic form, pelargonic acid vanillylamide or capsaicin II (PAVA), are highly concentrated forms of the active ingredients in hot peppers. They are available to the lay public in some countries as personal protective “pepper spray” and as military grade agent OC spray, but are not publicly available in the United Kingdom [19]. Agent OC is increasingly prevalent in crowd-control contexts and has been used on protesters globally [20–23]. While several countries have limitations on the possession and use of OC, it is unregulated in most countries [24, 25].

The volume and concentration of chemical in each spray and aerosol varies considerably among manufactures and countries [18]. Stated concentrations of OC may be misleading, because the potency of OC is dependent not only on the concentration within a solvent but on the strength of the capsicum extracted [20–22, 25]. Of concern, chemical irritants may contain numerous other toxic chemicals, including alcohols, organic solvents, halogenated hydrocarbons, and propellants such as Freon, tetrachloroethylene, and methylene chloride. The use of solvents such as tetracholoroethylene and methylene chloride may enable deeper skin penetration as well as larger quantities of irritant to be dissolved and dispersed, potentially exacerbating some of the effects attributed to pepper spray [7, 16, 21, 26, 27]*.* Dose levels for symptoms, toxic effects and lethal outcomes of CS and OC have not been well established. Studies suggest that even a very low (.003 mg/m3) concentration can lead to ocular irritation [7]. The dose of CS and OC in exposed individuals may be markedly increased by the use of multiple grenades and/or canisters at the same location over a short period of time, particularly in areas where people cannot easily escape. This further complicates the analysis of the toxicity of these chemicals in everyday use.

There is limited knowledge about the burden of injury from chemical irritants. There is also inadequate understanding of potential risk factors contributing to more severe injuries, as well as how law enforcement actions and policy may impact these injuries. While several recent reviews seek to better understand the range of injuries attributed to agent CS specifically [28] or the medical effects of several different agents [29], we know of no other review that seeks to provide data on injuries secondary to both agent CS and agent OC in the context of crowd control. To address some of the gaps in the literature and understand the burden of injury attributed to chemical irritants, as well as to better understand the role of law enforcement and policy makers, we conducted a systematic review of data on injuries, permanent disabilities, and deaths secondary to chemical irritants worldwide over the past 25 years. We sought to review the type and severity of injuries of individuals who present for medical care after exposure to chemical irritants, compare the impacts of agents used and study the factors that may have an effect on the rate and severity of injuries.

## Methods

We undertook a systematic review of the literature to determine the burden, severity, and range of injuries from chemical irritants using the Preferred Reporting Items for Systematic Reviews and Meta-analyses (PRISMA) guidelines.

### Search strategy

We searched PubMed, Toxnet, JSTOR, and Scopus using search terms cross-referenced with the MeSH database with no language restrictions [30]. We expanded our search to include non peer-reviewed publications as well as relevant reports identified by experts in the field. Gray literature searches were also conducted using reference lists of relevant articles and recommendations from experts. We included data from all types of studies, including experimental and observational studies and case series with at least five subjects.

In our search terminology, we tried to capture the diverse terms used for chemical irritants in the literature, including “tear gas,” “pepper spray,” and agents CS, CN, CR, CX, OC, and PAVA (Table 1). The databases and complete search terms are presented in the appendix. References were managed using the bibliographic software Zotero (V4.0.28.6).

**Table 1:** Keywords used for search

2-chloracetophenone	Less lethal weapons
blistering agent	Mace
blistering gas	noxious gas
capsaicin	O-chloronitrile
capsicum canister	OC gas
capsicum spray	OC spray
chemical agent	oleoresin capsicum
chemical weapons	PAVA
CN gas	pepper spray
CR gas	Phenacyl chloride
crowd control weapon	poison gas
CS gas	riot gas
gas rounds	riot spray
lacrimating agent	riot toxin
lacrimation agent	stink spray
lacrimation gas	tear gas
lacrimator gas	tear gas canister
less lethal	toxic gas

### Study selection

Articles were included if they documented injuries, deaths, or other medical or psychological health consequences of chemical irritants on human subjects and were published between January 1, 1990 and March 30, 2015. We included studies of cohorts of all ages, genders, and ethnicities. We included data from all contexts of chemical irritant use, including demonstrations and protests, riots, sporting events, prisons, arrests, and accidental exposures, as well as military or police training events. We excluded studies that lacked adequate documentation on injuries, were not accessible for full text review, or were animal and cadaver studies.

Titles and abstracts of the articles were screened for relevance. Full texts of all potentially relevant articles were reviewed against our inclusion criteria.

### Data extraction

Data from all eligible articles were then extracted and compiled in a database (Microsoft Excel for Mac 2011 v14.4.1). All articles were read and coded by two authors (RH and MD). Disagreements were resolved by discussion between the authors. For each study, we identified the chemical agent, deployment mechanism (spray versus aerosol or versus mechanical injuries from the projectile munition), region/country, demographic characteristics, and study setting. We categorized the outcome for subjects as recovered, permanently disabled, or dead, and classified each injury by severity and body system. Injury severity was coded based on the acuity and the resources required to manage that injury. Minor injuries were transient symptoms that may not be present on physical exam or are expected side effects of chemical irritants, such as blepharospasm, lacrimation, mild respiratory distress, sore throat, or nausea. Moderate injuries were those that were unexpected from previous published data on chemical irritants, were evident on physical exam, or lasted longer than expected, but may not require management by a health professional. Injuries such as persistent skin rashes or erythema, first-degree burns, conjunctivitis or eye injuries, oropharyngeal edema, persistent respiratory symptoms, and vomiting were classified as moderate injuries. We classified as severe injuries those that necessitate professional medical care, such as lacerations requiring sutures, second- or third-degree burns, airway obstruction, severe ocular trauma, cardiopulmonary disease, or abdominal injuries requiring medical or surgical management. Injury data was only included if it was documented by a medical professional. Injuries that were reported by patients, without any documentation, were excluded.

We utilized the NIH Quality Assessment Tool to classify each article as poor, moderate, or high quality [31]. This tool was chosen to standardize the quality of case series and observational studies, which made up a majority of the identified articles.

### Data analysis

We conducted descriptive analysis of injuries from chemical irritants to categorize the range of injuries and their severity. We also evaluated mediating or moderating environmental or practical factors that may have increased or reduced injuries. We expected significant heterogeneity and quality limitations that would preclude pooled data analysis or a direct comparison of the different chemical irritants.

## Results

**Fig. 1:**
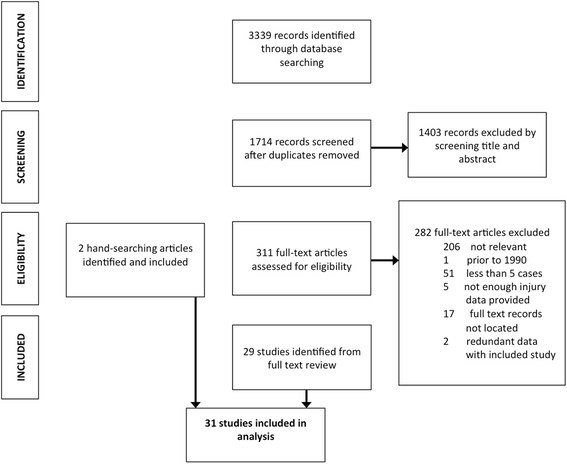
Study selection

Our search yielded 1714 discrete studies, of which 311 required full text review (Fig. 1). Twenty-nine articles met inclusion criteria and were included in our review (Table 2) [32–60]. Two additional articles and reports were identified by hand-searching the citation lists of included articles and by expert consultations [61, 62]. Quality assessment of the articles yielded 21 “high quality studies” that fulfilled 7 or greater of the nine criteria and 10 “moderate quality studies” that fulfilled between 4 and 6 of the criteria. (Table 2 is categorized by study quality).

**Table 2:** Study summaries

Reference	Country	Study Design	Context Type	Chemical Agent	Deployment Type	People Exposed	People Injured	Deaths	Perm Injuries	All injuries
High quality studies										
Adang, O.M.J., Mensink, J., 2004. Pepper spray: An unreasonable response to suspect verbal resistance. Policing 27, 206–219. doi:10.1108/13639510410536823	Netherlands	Prospective Cohort	Arrest/police duty	OC	hand-held spray	465	78	0	0	78
Anderson, P.J., Lau, G.S., Taylor, W.R., Critchley, J.A., 1996. Acute effects of the potent lacrimator o-chlorobenzylidene malononitrile (CS) tear gas. Hum Exp Toxicol 15, 461–465.	Hong Kong	Retrospective Cohort	Detention Center/Prison	CS	aerosol	184	184	0	0	391
Arbak, P., Başer, I., Kumbasar, Ö.O., Ülger, F., Kılıçaslan, Z., Evyapan, F., 2014. Long term effects of tear gases on respiratory system: analysis of 93 cases. ScientificWorldJournal 2014, 963,638. doi:10.1155/2014/963638	Turkey	Retrospective Cohort	Protest	CS and OC	aerosol	93	93	0	23	219
Breakell, A., Bodiwala, G.G., 1998. CS gas exposure in a crowded night club: the consequences for an accident and emergency department. J Accid Emerg Med 15, 56–57.	UK	Case Series	law enforcement use on non-protest crowd	CS	aerosol	23	11	0	0	15
Brown, L., Takeuchi, D., Challoner, K., 2000. Corneal abrasions associated with pepper spray exposure. Am J Emerg Med 18, 271–272.	USA	Retrospective Cohort	Arrest/police duty	OC	hand-held spray	100	47	0	0	47
Euripidou, E., MacLehose, R., Fletcher, A., 2004. An investigation into the short term and medium term health impacts of personal incapacitant sprays. A follow up of patients reported to the National Poisons Information Service (London). Emerg Med J 21, 548–552. doi:10.1136/emj.2003.012773	UK	Retrospective Cohort	multiple settings	CS	hand-held spray	152		0		319
Kearney, T., Hiatt, P., Birdsall, E., Smollin, C., 2014. Pepper spray injury severity: ten-year case experience of a poison control system. Prehosp Emerg Care 18, 381–386. doi:10.3109/10903127.2014.891063	USA	Retrospective Cohort	multiple settings	OC	hand-held spray	3671	3671	0	0	5261
Khan, S., Maqbool, A., Abdullah, N., Keng, M.Q., 2012. Pattern of ocular injuries in stone pelters in Kashmir valley. Saudi J Ophthalmol 26, 327–330. doi:10.1016/j.sjopt.2012.04.004	India	Retrospective Cohort	Protest	CS	projectile canister	2	2	0	2	2
Koul, P.A., Mir, H., Shah, T.H., Bagdadi, F., Khan, U.H., 2014. Effects of pepper grenade explosions on non-combatant bystanders. J Public Health Policy 35, 499–505. doi:10.1057/jphp.2014.15	India	Retrospective Cohort	Protest	OC	aerosol	294	294	0	0	1230
Lee, R.J., Yolton, R.L., Yolton, D.P., Schnider, C., Janin, M.L., 1996. Personal defense sprays: effects and management of exposure. J Am Optom Assoc 67, 548–560.	USA	Prospective Cohort	law enforcement training or activity	OC	hand-held spray	22	22	0	0	26
Nathan, R., Wood, H., Rix, K., Wright, E., 2003. Long-term psychiatric morbidity in the aftermath of CS spray trauma. Med Sci Law 43, 98–104.	UK	Retrospective Cohort	law enforcement use on non-protest crowd	CS	hand-held spray	30	23	0	14	23
Parneix-Spake, A., Theisen, A., Roujeau, J.C., Revuz, J., 1993. Severe cutaneous reactions to self-defense sprays. Arch Dermatol 129, 913.	France	Case Series	Arrest/police duty	CS	hand-held spray	11	11	0	5	25
Payne-James, J.J., Smith, G., Rivers, E., O’Rourke, S., Stark, M., Sutcliffe, N., 2014. Effects of incapacitant spray deployed in the restraint and arrest of detainees in the Metropolitan Police Service area, London, UK: a prospective study. Forensic Sci Med Pathol 10, 62–68. doi:10.1007/s12024-013-9494-7	UK	Prospective Cohort	Arrest/police duty	CS and OC	hand-held spray	99	93	0	0	319
Sharma, A.K., Shah, D.N., Shrestha, J.K., Thapa, M., Shrestha, G.S., 2014. Ocular injuries in the people’s uprising of April 2006 in Kathmandu, Nepal. Nepal J Ophthalmol 6, 71–79. doi:10.3126/nepjoph.v6i1.10775	Nepal	Retrospective Cohort	Protest	CS	projectile canister	3	3	0	2	3
Thomas, R.J., Smith, P.A., Rascona, D.A., Louthan, J.D., Gumpert, B., 2002. Acute pulmonary effects from o-chlorobenzylidenemalonitrile “tear gas”: a unique exposure outcome unmasked by strenuous exercise after a military training event. Mil Med 167, 136–139.	USA	Retrospective Cohort	law enforcement training or activity	CS	aerosol	38	9	0	0	45
Vesaluoma, M., Müller, L., Gallar, J., Lambiase, A., Moilanen, J., Hack, T., Belmonte, C., Tervo, T., 2000. Effects of oleoresin capsicum pepper spray on human corneal morphology and sensitivity. Invest. Ophthalmol. Vis. Sci. 41, 2138–2147.	Finland	Prospective Cohort	law enforcement training or activity	OC	hand-held spray	10	10	0	0	51
Wani, A.A., Zargar, J., Ramzan, A.U., Malik, N.K., Qayoom, A., Kirmani, A.R., Nizami, F.A., Wani, M.A., 2010. Head injury caused by tear gas cartridge in teenage population. Pediatr Neurosurg 46, 25–28. doi:10.1159/000314054	India	Prospective Cohort	Protest	CS	projectile canister	5	4	1	1	4
Wani, M.L., Ahangar, A.G., Lone, G.N., Singh, S., Dar, A.M., Bhat, M.A., Ashraf, H.Z., Irshad, I., 2011. Vascular injuries caused by tear gas shells: surgical challenge and outcome. Iran J Med Sci 36, 14–17.	India	Prospective Cohort	Protest	CS	projectile canister	18	18	0	13	50
Watson, K., Rycroft, R., 2005. Unintended cutaneous reactions to CS spray. Contact Derm. 53, 9–13. doi:10.1111/j.0105-1873.2005.00585.x	UK	Retrospective Cohort	law enforcement training or activity	CS	hand-held spray	7	7	0	3	8
Watson, W.A., Stremel, K.R., Westdorp, E.J., 1996. Oleoresin capsicum (Cap-Stun) toxicity from aerosol exposure. Ann Pharmacother 30, 733–735.	USA	Retrospective Cohort	Arrest/police duty	OC	hand-held spray	94	80	0	0	192
Zollman, T.M., Bragg, R.M., Harrison, D.A., 2000. Clinical effects of oleoresin capsicum (pepper spray) on the human cornea and conjunctiva. Ophthalmology 107, 2186–2189.	USA	Prospective Cohort	law enforcement training or activity		OC Spray	47	47	0	0	208
Moderate Quality Studies										
Atkinson, H., Sollom, R., 2012. Weaponizing Tear Gas: Bahrain’s. Physicians for Human Rights, Boston, MA.	Bahrain	NGO Report	Protest	CS	aerosol	11	10	1	3	9
Dong, C., de la Garza, A., 2007. Chlorobenzylidenemalonitrile gas exposure from a novelty personal-protection gun. Cal J Emerg Med 8, 57–60.	USA	Case Series	Accidental exposure	CS	aerosol	8	8	0	0	16
Hankin, S.M., Ramsay, C.N., 2007. Investigation of accidental secondary exposure to CS agent. Clin Toxicol (Phila) 45, 409–411. doi:10.1080/15563650701285438	UK	Case Series	Accidental exposure	CS	aerosol	34	21	0	0	147
Karagama, Y.G., Newton, J.R., Newbegin, C.J.R., 2003. Short-term and long-term physical effects of exposure to CS spray. J R Soc Med 96, 172–174.	UK	Retrospective Cohort	law enforcement use on non-protest crowd	CS	hand-held spray	34	34	0	0	109
Kiel, A.W., 1997. Ocular exposure to CS gas: the importance of correct early management. Eye (Lond) 11 (Pt 5), 759–760. doi:10.1038/eye.1997.194	UK	Case Series	Accidental exposure	CS	aerosol	6	6	0	0	6
Oh, J.J., Yong, R., Ponampalam, R., Anantharman, V., Lim, S.H., 2010. Mass casualty incident involving pepper spray exposure: Impact on the emergency department and management of casualties. Hong Kong Journal of Emergency Medicine 17, 352–359.	Singapore	Retrospective Cohort	Accidental exposure	OC	hand-held spray	13	11	0	0	38
Rasier, R., Kukner, A.S., Sengul, E.A., Yalcin, N.G., Temizsoylu, O., Bahcecioglu, H.O., 2014. The Decrease in Aqueous Tear Production Associated with Pepper Spray. Curr. Eye Res. 1–5. doi:10.3109/02713683.2014.930156	Turkey	Retrospective Cohort	Protest	OC	hand-held spray	96	25	0	0	25
Solomon, I., Kochba, I., Eizenkraft, E., Maharshak, N., 2003. Report of accidental CS ingestion among seven patients in central Israel and review of the current literature. Arch. Toxicol. 77, 601–604. doi:10.1007/s00204-003-0479-2	Israel/Palestine	Case Series	Accidental exposure	CS	aerosol	7	7	0	0	37
Unuvar, et al. 2013. HRFT	Turkey	NGO Report	protest	CS	aerosol	269	247	0	0	266
Unuvar, U., Ozkalipci, O., Irencin, S., Sahin, U., Fincanci, S.K., 2013. Demonstration control agents: evaluation of 64 cases after massive use in Istanbul. Am J Forensic Med Pathol 34, 150–154. doi:10.1097/PAF.0b013e3182887b3c	Turkey	Retrospective Cohort	Protest	CS and OC	aerosol	64	53	0	0	88

### Demographic analysis

**Fig. 2:**
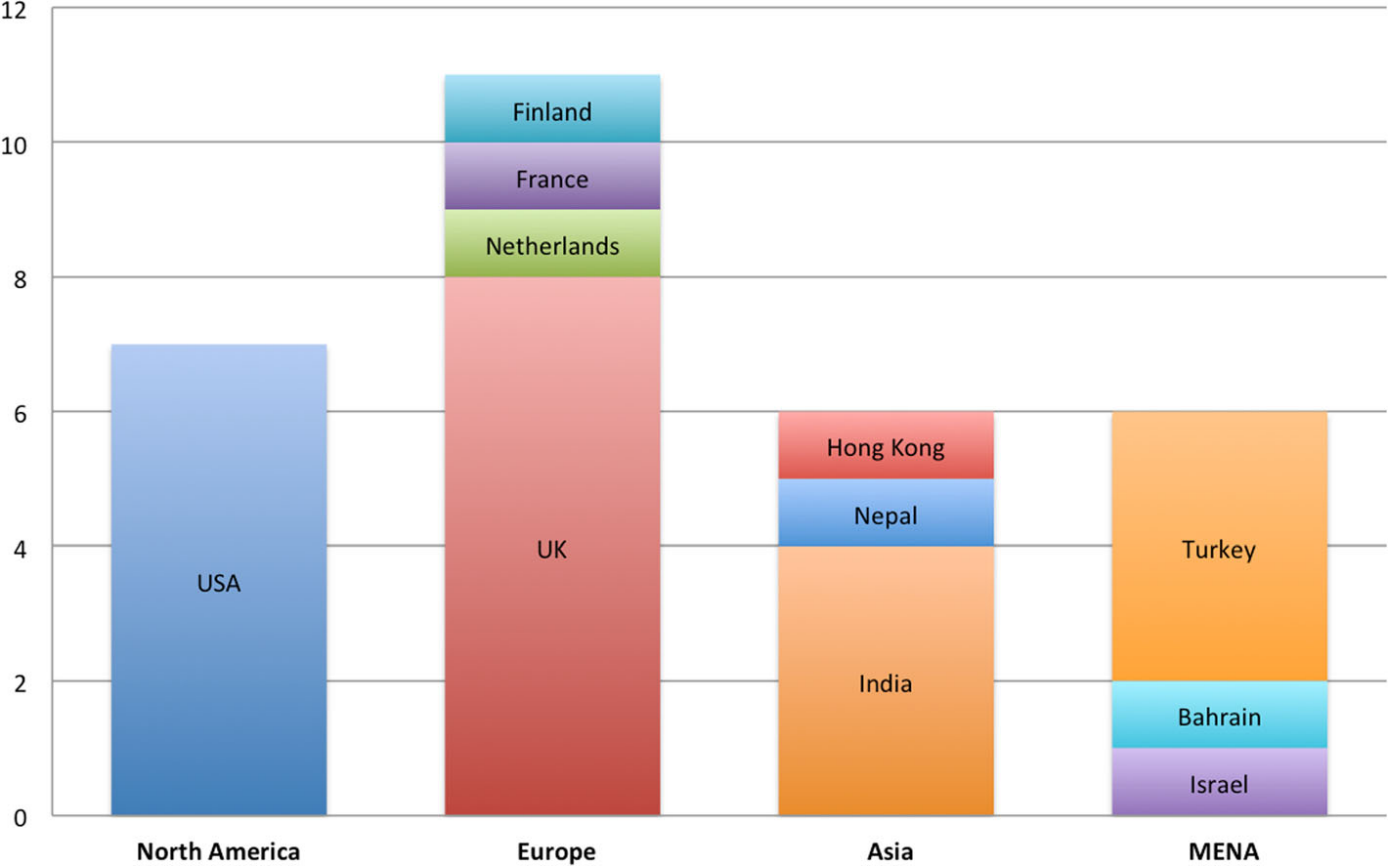
Region and country of included studies

Of the total of 31 studies included in the analysis, 16 were retrospective cohort studies, seven were prospective cohort studies, six were case series and two were non-peer reviewed reports from reputable human rights organizations. The number of subjects ranged from two to 3697 (median 31) (one study met the inclusion criteria of five subjects, and although several of the subjects sustained injuries from another weapon, the study was nonetheless included). In studies in which gender was reported, 57% of subjects were male and 43% were female. In studies in which age was reported, the age ranged from 3 months to 94 years, with an mean age of 25.7 years. The injury context included protests (10), arrests (five), military or police training exercises (five), accidental exposures (five), and a detention center riot (one); some of the studies included injuries in more than one context. The eligible studies included data from 11 countries and were published between 1993 and 2015 (Fig. 2).

Twenty-six studies included data on injuries caused by Agent CS and 14 included data on injuries caused by Agent OC. Sixteen studies evaluated dry aerosolized forms, such as grenade-type deployment of the chemical agent and 15 included sprays formulated with solvents. Seven of the studies recorded injuries that resulted from the projectile munition containing chemical irritant causing direct trauma to subjects.

### Analysis of injuries and deaths

In the included articles, a total of 5910 people were exposed to chemical irritants and sought medical attention, of whom 5131 (87%) suffered injuries or died as a result of the exposure. Of those who suffered injuries, two people died and 67 (1.3%) suffered permanent disability. The majority fully recovered from their injuries (98.7%).

### Deaths

Two deaths were documented in the selected articles. A report from Bahrain documented the case of a man who died of respiratory arrest after agent CS aerosol was fired inside his home. In another case, the chemical irritant projectile munition contributed to one death from traumatic brain injury after protests in Nepal. There were no deaths associated with agent OC.

### Permanent injuries and disabilities

**Fig. 3:**
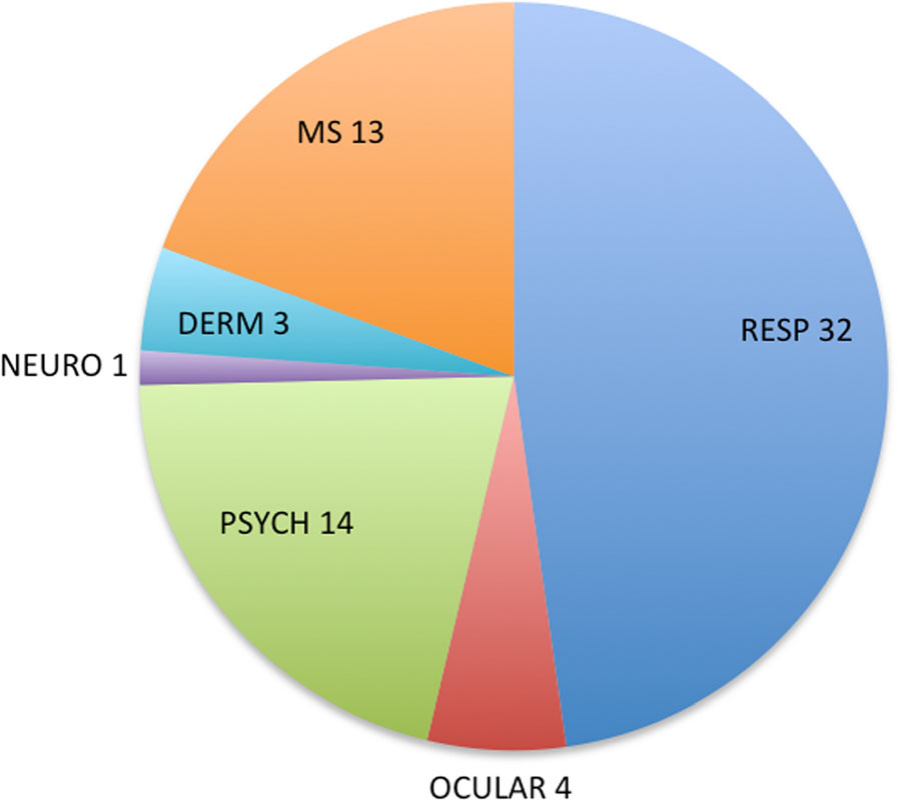
Permanent injuries from chemical irritants

Fifty-eight people experienced permanent disability (Fig. 3). Eighteen of the disabilities were secondary to traumatic injuries from the projectile munitions. These included globe ruptures and blindness (four people), traumatic brain injury resulting in a persistent vegetative state (one person), limb amputations (three people), and functional loss of limbs (10 people). Persistent psychiatric symptoms were documented in 14 people and persistent symptoms of asthma and other respiratory complaints were reported in 23 people. Chronic dermatological conditions such as hypersensitivity reactions were documented by skin testing in three people. In one study of 297 individuals seeking care and/or evaluation of injuries following the 2013 Gezi Park protests in Turkey, 117 psychiatric evaluations were conducted. Of those, 50 (43%) met diagnostic criteria for acute stress disorder, 27 (23%) met diagnostic criteria for post-traumatic stress disorder (PTSD), and nine (8 %) met diagnostic criteria for major depressive disorder [62].

### Injuries

**Fig. 4:**
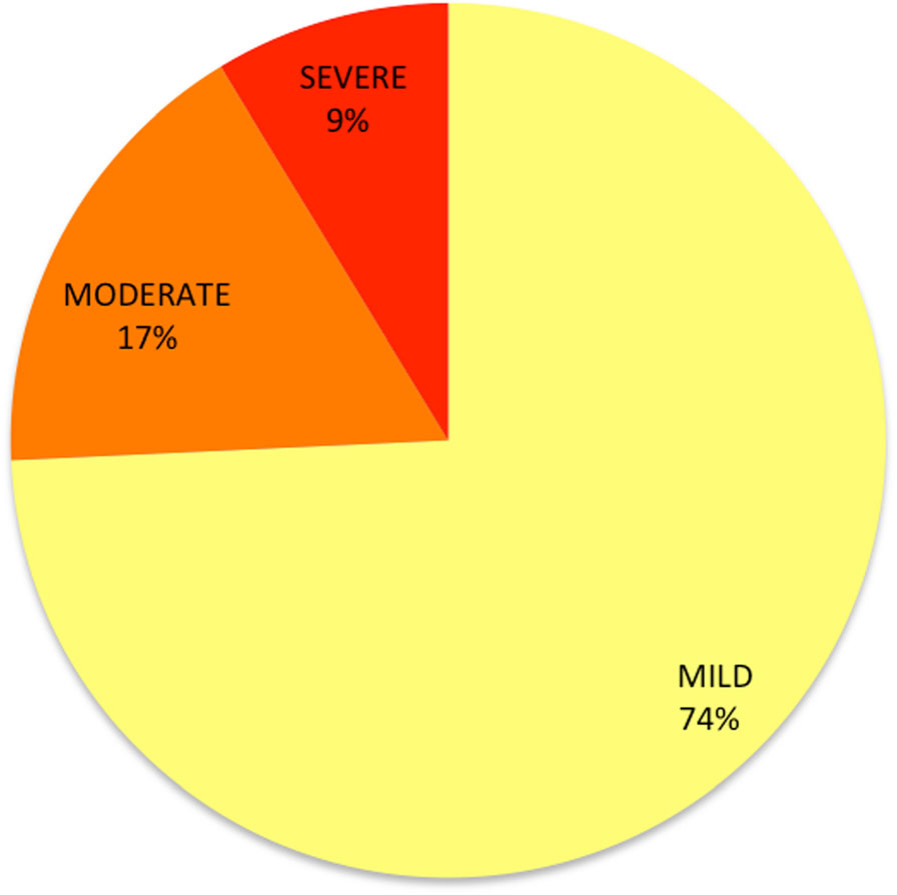
Injury severity from chemical irritants

**Fig. 5:**
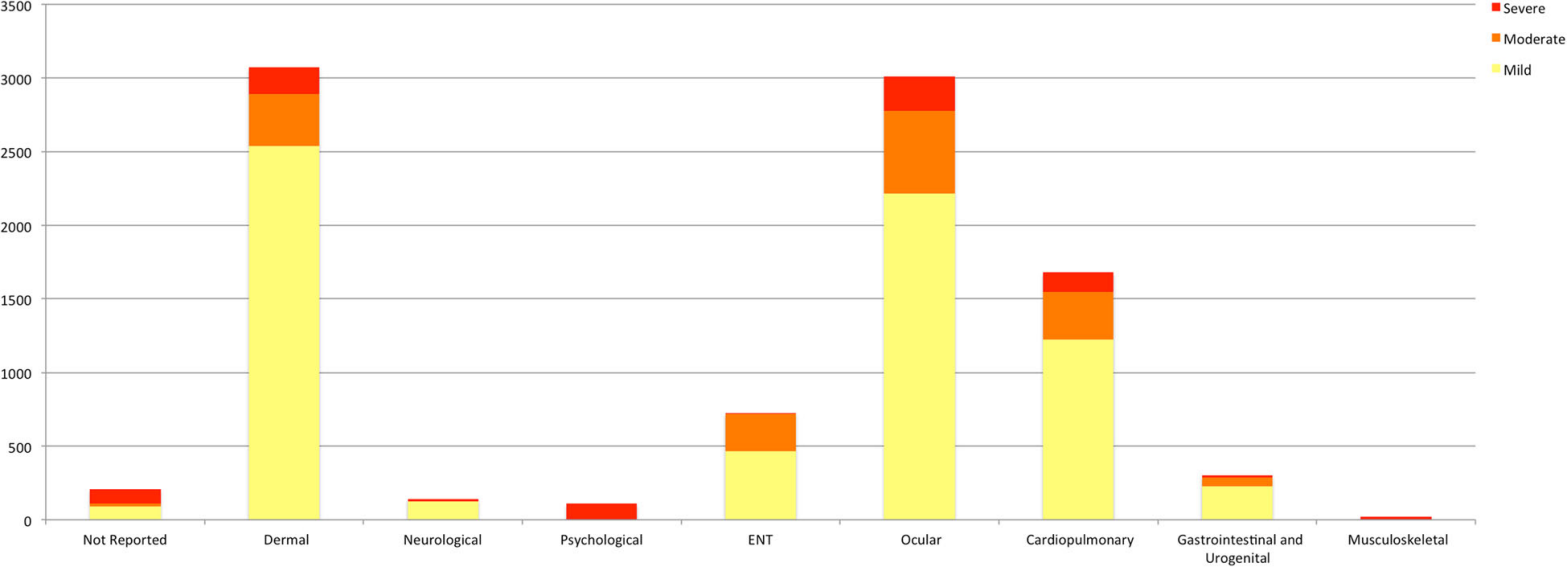
Injury severity by body system

There were 9261 documented injuries, with multiple injuries occurring in each individual. In total, 6878 (74.2%) of the injuries were categorized as mild, 1582 (17%) were moderate injuries, and 865 (8.7%) were severe injuries (Fig. 4). While many body systems were affected, the majority of injuries were to the skin, eyes, and cardiopulmonary system. Injury severity varied depending on body system affected (Fig. 5). Severity followed a pattern of fewer injuries among the higher severity categories for dermal, ocular, neurological, cardiopulmonary, and intra-abdominal injuries. For instance, while the majority of skin injuries (2539) were mild, 351 of the injuries were moderate and 180 were severe. Similarly, for cardiopulmonary injuries, there were 1220 mild injuries, 328 moderate injuries and 131 severe injuries. On the other hand, there were more severe neurological injuries (12%) than moderate neurologic injuries (1 %). Injuries to the musculoskeletal system, as well as psychological injuries, were all categorized as severe, based on the definition employed.

### Chemical agent

**Fig. 6:**
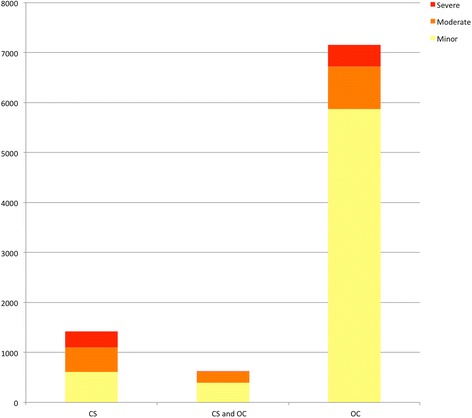
Injuries by chemical agent

The nature of chemical irritant exposure and injuries is also related to the chemical agent used, mechanism of deployment, environmental conditions, and context of use. Fourteen studies dealt exclusively with agent CS and 10 studies exclusively studied agent OC. Three studies included injury data from both chemical agents or did not differentiate between the two. Four studies reported exclusively on traumatic injuries from the projectile munition, while three other studies reported some injuries from the projectile munition among other injuries from the chemical agents themselves. Among 7156 documented injuries specifically from agent OC, only 6 % were categorized as severe. In contrast, 27.9% of 1148 injuries from agent CS were categorized as severe (Fig. 6).

### Other factors that may impact injury severity

To assess for other factors that may impact injury severity, we utilized a qualitative approach by perusing the articles for data that may not fit into categorical variables but would regardless be relevant. Several of these factors were noted in the article text and are highlighted.

### Deployment mechanism

The included studies documented injuries secondary to both dry aerosolized and soluble spray forms of both chemicals. Proximity to the area where the chemical was released and the force of the propellant affected outcomes [38, 40, 47, 53, 60]. We identified 5366 mild injuries, 884 moderate injuries, and 483 severe injuries from spray forms of agent CS and OC. Among the injuries from spray forms of chemical irritants, 7.2% were severe. We documented 1512 mild injuries, 676 moderate injuries, and 281 severe injuries from aerosolized forms of agent CS and agent OC. Among injuries from aerosolized forms, 11.6% were severe. Comparative analysis of the deployment mechanisms using pooled data was not conducted, given the concern for confounding factors.

The use of projectile munitions was documented to cause 231 injuries, of which 63 (27%) were severe. There were 73 traumatic injuries to the head and neck, including at least four people who lost vision in an eye due to projectile munition trauma. We documented 45 injuries to the torso (chest, abdomen, back, and genitalia). There were 61 upper extremity injuries and 34 lower extremity injuries (including at least three people requiring amputations and 10 with severe functional loss of a limb due to neurovascular injuries). Eighteen dermal injuries (8 %) included bruises, lacerations, and heat burns.

### Other factors

Several other factors were documented as exacerbating the potential for injury, but they lacked detailed data for analysis, such as documentation of specific injuries. Utilizing the weapons in confined spaces and in areas where people could not easily escape potentially increased the exposure to the irritant either in quantity or over time [54]. One study in a detention center suggested that the excessive number of injuries may have been caused by the crowded and enclosed setting, which offered no opportunity for people to escape [33]. Use of chemical irritants in areas with high heat or humidity potentially exacerbated skin irritation, and windy conditions risked the contamination of law enforcement officers, bystanders, or nearby residences and businesses [33, 43, 53]. One study noted that the use of agent CS for military training on a particularly humid day, followed by strenuous exercise by trainees, may have caused severe respiratory injuries which resulted in several people requiring ICU-level care [53]. Direct targeting of the face and eyes by hand-held spray has been noted to cause trauma and toxicity to the cornea and conjunctiva of the eye [40, 41, 45, 50].

## Discussion

The findings of our systematic review identified significant morbidity and mortality associated with chemical irritant agents CS and OC. Chemical irritants, like many other crowd-control weapons, are typically justified as a safe tool to disperse potentially dangerous groups or incapacitate threatening individuals as part of the effort to ensure public safety. The prevailing presumption about these chemical agents is that they cause minimal and transient irritation to the skin and eyes, but are generally safe for use on diverse populations. However, we found that, by design or by inappropriate use, chemical irritants can cause significant injuries as well as permanent disabilities. While deaths were rare, we identified one death directly caused by the blunt trauma from the projectile and another from high dose exposure to the chemical agent in a closed environment. These health consequences may be related to the chemical agents themselves, the total exposure dose, the deployment technique, or the way these weapons are used in different settings.

Our findings indicate that agent CS and agent OC were used both in protests and during arrest scenarios and training exercises; contact also occurred through accidental exposure. Chemical irritants caused injuries to many different body systems in addition to the expected pain to the skin and eyes. We also documented a range of injury severity for neurological, oropharyngeal, cardiac, pulmonary, and musculoskeletal systems. The psychological impact of the use of crowd-control weapons has not been well studied or documented in the medical literature, but cases described in this review indicate that exposure to CCWs may result in significant psychiatric symptoms and long-term disability.

In addition to documenting injuries, we identified factors that may impact injury severity. Intrinsic characteristics of the chemical agents themselves play a role. Chemical irritants, especially those deployed in aerosolized forms, are inherently indiscriminate and can affect not only the intended targets but also peaceful demonstrators, bystanders, nearby communities and residences, and law enforcement officers themselves. The majority of people injured are young adults, consistent with typical protest demographics [63, 64]. We found a relatively equal gender distribution of injuries. But many studies also found injuries among children and elderly people that appear to validate concerns about the indiscriminate nature of chemical irritants and their potential impact on bystanders and nonviolent demonstrators [65]. Children are more vulnerable to severe injuries from chemical toxicity [66, 67]. The elderly and those with chronic diseases are also prone to worse outcomes from chemical irritants [68, 69]. Because of the indiscriminate nature of chemical irritants, limiting the exposure to individuals or small groups is difficult. Most often a large, diverse, and differentially susceptible group will be exposed, posing the risk of unnecessarily injuring nonviolent, potentially vulnerable people.

Perhaps even more concerning are the effects of these chemical agents in settings where people are chronically exposed to these chemicals, either by repeated use near their homes or businesses, or because of occupational use in which safety has never been studied and cannot reasonably be assumed [34, 55, 58]. Repeated exposure may be particularly concerning for law enforcement officers, people who attend protests frequently, and health workers who may experience multiple occupational exposures.

The decision to use chemical agents in specific environmental conditions and social contexts may also play a role in injury severity. Clinical effects are likely dose-dependent and excessive exposure may exacerbate severity. Studies included in this review show that the use of chemical irritants in enclosed spaces without safe avenues of egress increases exposure to the agent and exacerbates ensuing injuries [33, 35, 37, 39, 40, 61]. Although our study excluded secondary injuries, we note reports that there were several cases of chemical irritants sparking mass panic and stampedes that contributed to significant morbidity and mortality. These include at least 20 deaths in a sports stadium in Egypt in 2015, [70] 15 deaths in the Democratic Republic of the Congo in 2014, [71] 11 deaths in a stampede in Zimbabwe in 2014, [72] and 43 in South Africa in 2001, [73] all during protests or in other crowded contexts. Deliberately aiming the munition as a projectile weapon into dense crowds or at individuals can cause severe traumatic injury [42, 51, 54, 57, 62].

In conducting the broader research, we also identified significant public concern over lack of transparency by law enforcement and manufacturers about the agent(s) used during specific events. Manufacturers often do not provide adequate information on concentrations of chemicals or the solvents and non-active ingredients that may contribute to toxicity. In addition to the difficulties this may pose to health workers trying to appropriately manage patient injuries, lack of transparency can break down trust and negatively impact relationships between communities and law enforcement. Though this may be an intended outcome in some repressive regimes, we noted this concern in all uses of toxic chemical agents against primarily unarmed civilian populations.

### Policy implications and recommendations

The legal protections of the rights to freedom of expression and peaceful assembly, along with general principles on the proportionate use of force by law enforcement, provide some general guidance that the use of chemical irritants, along with other crowd-control weapons, should be limited. Specifically, CCWs should only be used in situations where particular individuals pose an imminent violent threat, or where a protest requires dispersal because of widespread violent acts that pose an imminent threat to public safety [74, 75]. In most situations where we found these weapons being used, neither of these conditions was documented. The use of chemical irritants as crowd-control weapons must be considered in the broader context of human rights, public safety, use of force, and law enforcement practices necessary to maintain order in the context of demonstrations. Open communication with demonstrators and the community, arrests of violent individuals, and safeguards for legal demonstration may obviate much of the demand for these chemicals. Given the frequency of serious injury, disability, and death, the use of chemical irritants should be strictly limited to situations of imminent harm that cannot be policed effectively with safer methods.

The Chemical Weapons Convention (effective since 1997 with 192 state signatories excluding only Egypt, Israel, North Korea and South Sudan) prohibits the use of these riot control agents’ during warfare. Significant questions exist on the legality of military use of these weapons in civilian protest. We recommend that law enforcement and the military be obligated to maintain transparent and accurate data on use-of-force incidents, particularly those that employ chemical irritants and other crowd-control weapons. Active surveillance of injuries caused by chemical irritants is vital for manufacturers, law enforcement, and the community in order to understand the risks and dangers of these weapons. This data should be available and accessible to the public for independent analysis.

We also note that combinations of OC and CS are becoming more common, both in spray and aerosol forms as well as within projectiles such as the “pepper ball” [16, 76, 77]. Several newer agents are also in development, including agents CS1 and CS2 (which may extend the half-life of CS or facilitate higher dermal penetration) and agent CX, which is reported to be more potent than agent CS [16]. Each of these potential enhancements to weapons may compound the already large number of injuries. It is important that we address the human costs of current chemical irritants before developing new, more potent ones.

While making law enforcement protocols publicly available may not be possible due to security risks, we recommend that police and military departments make every effort to communicate with health workers and the community in order to minimize potential injuries from chemical irritants and to maintain trust. Training of police officers must include education on human rights principles and the obligation of the police to protect peaceful protestors. Police should also be trained in the dangers of chemical irritants, guidelines on the safe utilization of chemical irritants, the risks of repeated exposures, environmental factors, and the risks of direct trauma from poorly-aimed projectile munitions, as well as other risk factors.

### Limitations

Our systematic review had several limitations. In the absence of systematic reporting requirements on deaths and injuries in crowd-control settings, it is likely that we have largely underestimated the prevalence of deaths and injuries. The limited follow-up in many of the articles also highlighted the lack of data on the chronic health impacts of these weapons, which are likely underreported. We note that in our attempt to ensure valid and reliable injury data, we have not accounted for a large number of injuries and deaths from chemical irritants that have been reported by the news media, by social justice organization reports, and by social media, many of which include photographic or videographic evidence of injuries. We also excluded reports that lacked injury specifics or clear causation from chemical irritants, but were likely linked to chemical irritant utilization. We also excluded the significant number of case reports in the published literature to avoid biasing our results towards the publication of the most severe injuries. In addition, there is wide variability in how weapons are used and in the specific concentrations of chemical agents; there is also a lack of data on the number of people exposed. Given these concerns, we were not able to calculate population estimates of the impact of chemical irritants or compare any specific agents or numerical study results. However, severe injuries from chemical irritants are not rare or isolated incidents. They have occurred in many nations and under different types of regimes and law enforcement protocols.

This review was also limited by the quality and methodology of the available literature on chemical irritant injuries. There are several potential biases, including the potential over-publication of the most dramatic incidents and independent limitations on individuals’ decision to seek medical care. On the other hand, difficulty in gathering and publishing data in repressive regimes may limit the availability of injury data from many instances of chemical irritant utilization. There was also significant heterogeneity in the participants and medical treatments in different regions and clinical settings. There was considerable methodological variability in the study designs and settings. Each setting had variable standards on the use of chemical irritants. However, the overall quality of the studies was comparable to observational and case series-type studies addressing chemical irritants. Given the multiple confounding factors, we could not compare the chemical agents or deployment mechanisms. Included studies did not provide enough data to reliably estimate the risk of injury from any given chemical irritant in an exposed population.

## Conclusion

We found that chemical irritants cause severe injury, permanent disabilities, and in rare cases, death. Despite chemical irritants being recognized as safe weapons to disperse or control crowds, the number and types of injuries documented in this review highlight the serious risks associated with the frequent use of these weapons. Specific risks include the use of chemical irritants in enclosed spaces, excessive quantity of chemicals used, specific environmental factors such as heat and humidity and direct targeting of individuals, both with the projectile canister as well as spray to the face. Protocols to limit indiscriminate use of chemical irritants are urgently needed in order to safeguard human rights and prevent unnecessary morbidity and mortality among protestors and bystanders worldwide.

## Abbreviations

CCW: Crowd control weapon
CN: Chloroacetophenone
CS: 2-chlorobenzalmalonitrile
CX: Phosgene oxime
ICU: Intensive Care Unit
INCLO: International Network of Civil Liberties Organizations
NIH: National Institutes of Health (USA)
OC: Oleoresin capsicum
PAVA: Pelargonic acid vanillylamide or capsaicin II
PHR: Physicians for human rights
